# An auto-ethnographic study of co-produced health research in a patient organisation: unpacking the good, the bad, and the unspoken

**DOI:** 10.1186/s40900-024-00609-8

**Published:** 2024-07-23

**Authors:** Astrid Janssens, Danielle Drachmann, Kristy Barnes-Cullen, Austin Carrigg, Henrik Thybo Christesen, Becky Futers, Yvette Ollada Lavery, Tiffany Palms, Jacob Sten Petersen, Pratik Shah, Paul Thornton, Joseph Wolfsdorf

**Affiliations:** 1 Julius Center for Health Sciences and Primary Care, Bioethics & Health Humanities, University Medical Center Utrecht, Utrecht, The Netherlands; 2 Department of Public Health, User Perspective and Community-Based Interventions, University of Southern Denmark, Odense, Denmark; 3 Centre for Research with Patients and Relatives, Odense University Hospital, Odense, Denmark; 4 Ketotic Hypoglycemia International, Skanderborg, Denmark; 5 Patient-Centered Research, Evidera, London, UK; 6 Department of Clinical Research, University of Southern Denmark, Odense, Denmark; 7 Hans Christian Andersen Children’s Hospital, Odense University Hospital, Odense, Denmark; 8 Medical Ethics Committee, UCI Health, Orange, CA USA; 9 Novo Nordisk, Dicerna, Lexington USA; 10 The Royal London Children’s Hospital, Barts Health NHS Trust, London, UK; 11 Cook Children’s Medical Center, Fort Worth, Texas USA; 12 Boston Children’s Hospital, Harvard Medical School, Boston, Massachusetts USA

**Keywords:** Patient and public involvement and engagement, Auto-ethnography, Citizen science, Patient organisation, Rare disease

## Abstract

**Background:**

In rare diseases, limited access to services and rare disease experts may force families to act as medical advocates for their child; they can volunteer to support clinician-initiated research or initiate and lead research themselves. Ketotic Hypoglycemia International (KHI) is a new, global organization for families affected by idiopathic ketotic hypoglycemia (IKH) and is run solely by volunteers. Doing research together, families and international experts in a collaborative process such as at KHI, also referred to as patient and public involvement and engagement (PPIE) or extreme citizen science, is often praised for its positive effects on the research and the stakeholders involved.

**Methods:**

We used auto-ethnographic narratives from parents and medical professionals in KHI to report on their experiences with co-produced health research. All co-authors wrote down their experiences in relation to three topics: time invested, work invested and power dynamics.

**Results:**

Whilst the parents and health care professionals felt a new hope for (their) children with IKH, they also felt pressure to contribute time or to be flexible in how and when they dedicated time towards the organization. The power dynamics were characterised by a change in the relationship between the parents and medical experts; the parent being taught by the expert shifted to the expert learning from the lived experience of the parent. Both parents and medical experts struggled with maintaining boundaries and safeguarding their mental health.

**Conclusion:**

Our findings call for the need to secure and prioritize funding for patient organizations, to enable them to create the sustainable architecture required for meaningful PPIE within these organizations. The morals and often deeply personal reasons for engaging with voluntary work in health research, can lead to overstepping of boundaries. As a result of our research, we call for the development of ethics of care guidelines within collaborative health research.

**Supplementary Information:**

The online version contains supplementary material available at 10.1186/s40900-024-00609-8.

## Abstract (plain language summary)

When confronted with a rare disease it is often hard to access information and or medical experts for help. Parents of children affected by idiopathic ketotic hypoglycemia (IKH) have joined in a patient-led organisation to initiate and lead research that could give answers to their medical questions and worries. Medical experts have been invited to join the organisation as members of the Scientific Advisory Board (SAB). When people report on health research conducted in collaboration with patients and or members of the public, they mostly mention positive outcomes. At KHI, some people had left the organisation and we had to deal with some difficult situations; so, we wanted to document and understand these challenges. Nine members of KHI, parents and medical experts, wrote down their stories, using three topics to guide their narrative: time invested, work invested and power dynamics at KHI. Parents and medical experts felt a new hope for (their) children with IKH when working for KHI but they also felt pressured to work at all hours and at the cost of time with their families or their own health. The stories revealed that parents felt less important compared to medical experts, but also that the relationship between parents and experts changed from the parent being taught by the expert, to the expert starting to learn from the lived experience of the parent. To make these collaborations successful we plead for funding for patient-led organisation and ethical guidelines to safeguard volunteers (both medical and lay people).

**Supplementary Information:**

The online version contains supplementary material available at 10.1186/s40900-024-00609-8.

## Introduction

There is a growing interest and uptake of patient and public involvement and engagement (PPIE) in health services and research. A significant growth in publications about lived experience engagement in health research is noted in the past decade [1–3], and an increasing number of national funding organisations and international journals endorse and promote patient and public involvement and engagement (PPIE) in research processes [4, 5]. It has recently been called “a moral, methodological, and policy imperative” ([6], p 1). The moral imperative refers to normative arguments for PPIE reflecting ethical and/ or political concerns with PPIE being considered an end in itself relating to values such as rights, justice, fairness and democracy [7]. The consumerist approach on the other hand, turns PPIE in a technical matter, is conservative and top-down, and interested in achieving instrumental goals through the inclusion of service users. In a quest to improve research quality and effectivity, address research waste, and produce research findings that address patient and public concerns, PPIE was stripped from its ideological and political relations. With PPIE a means to an end, the call for evidence has received great attention: the international literature evaluating the impact of PPIE has more than tripled in the last decade [8].

Two decades of PPIE have resulted in studies reporting evidence of (mainly) positive impact on the research process: patients and the public prioritise topics for research that are different to those of academics and health professionals [9], and James Lind Alliance and other patient prioritisation efforts have led to public funding calls using patient research questions [10]; patients’ contributions improve clinical trial design and material used in trials [11] resulting in more effective recruitment, response rates, enrolment and retention [12, 13]; investments in PPIE lead to cost savings due to the time saved getting a drug to the market [14]; and PPIE can influence what research outcomes are measured as well as how they are measured [15] making research findings more relevant.

In sum, PPIE is largely reported as ‘being unquestionably a good thing’ [16] as suggest the published studies evaluating the impact of PPIE [13, 17]. Critical reviews on co-produced health research have, however, raised questions about report bias or completeness of the evaluations in the majority of studies [16, 18, 19]. The PIRICOM study, dating from 2010, [20], was the first to extensively review and synthesise available evidence of impact of PPIE focusing on several areas, including effects on patients, researchers, and the research process. They only identified a few studies that reported negative impacts such as feelings of being overburdened, unheard, frustrated and marginalized. A more recent scoping review [21] confirmed a seemingly unbalanced reporting on PPIE outcomes and impact. Seventy papers reporting, reflecting, or evaluating collaborative health research were reviewed; all reported that the involvement activities ultimately resulted in positive changes in the projects. A few articles mentioned how the researchers were worried that the PPIE in their research project would decrease scientific rigour [11], not be taken seriously [22], or fail to obtain legitimacy amongst clinicians [23].

Recently, Richards and colleagues [24] made a refreshing contribution to the booming literature on PPIE. They presented four cases of what they call “patient engagement gone wrong”: patient partners as a check mark, unconscious bias towards patient partners, lack of support to fully include patient partners, and lack of recognizing the vulnerability of patient partners. Whilst we acknowledge - through personal experience - that partnerships and the process of engagement can go awry, in this paper, we report on a different kind of “engagement gone wrong”: the adverse effects of extreme citizen science or patient and public driven health research. Adverse effects – likely unwanted and unintended - on the professional and or personal lives of those involved in the collaborative health research process is unreported or unpublished and called for by Russell et al. [16] as part of their critical research agenda for patient involvement asking questions about possible harms of public involvement.

Ketotic Hypoglycemia International (KHI) is a global patient-led, non-governmental organization (NGO), founded by Danielle Drachmann (first author) in 2020, and hosts a network for families affected by the rare condition, idiopathic ketotic hypoglycemia (IKH). In short, the pathological variant of IKH leads to attacks of low blood glucose with accelerated fatty acid oxidation and ketone production, causing a range of manifestations including tremor, lethargy, altered behaviour, reduced consciousness, nausea, and vomiting [25]. KHI aims to enhance the understanding of IKH for the benefit of patients, families and caregivers, and to help educate the medical community regarding the condition. Early in its inception, parents asked medical experts to join the organisation as members of the Scientific Advisory Board (SAB).

In their quest for answers and from the conversations amongst and between the families and the medical experts, the families of KHI initiated literature searches and research projects to identify and fill gaps in medical literature. This interactive and iterative process of exchanges of lived experiences of a patient community, available medical literature and medical experts’ clinical experience, resulted in research projects that can be labelled as *extreme citizen science*, level 4 (the highest level) in Haklay’s taxonomy of citizen science [26] or co-production [27, 28]. Extreme citizen science and co-production, are characterized as collaborative science, where professional and non-professional scientists collaborate on the problem definition, data collection and analysis. These collaborative efforts have so far led to three novel scientific publications authored by family-caregivers and leading medical experts [25, 29, 30].

Over the years, KHI grew organically into a non-profit organization currently reaching over 1800 affected families, run entirely by volunteers – both parents as well as medical experts. During this exponential growth, the organization suffered growing pains managing a continuously increasing workload and interest from both patients, families and stakeholders from the medical field (e.g. pharmaceutical industry). The resignation of four core members of KHI and life-changing events in the personal and professional lives of some of the core members of KHI triggered some volunteers to critically reflect on the objectives of KHI, the work demand and production, the volunteers’ interactions, their contribution to KHI, and how working for a recently founded, fast growing non-profit organization affected them. Questions raised included: what are the consequences of being invested in an international non-governmental organization across clinical and academic borders; what are the personal and professional implications and or consequences; what can we expect from ourselves and others; how and where to draw boundaries and look after ourselves and KHI colleagues / friends? We gathered these introspective thoughts in a collection of auto-ethnographic narratives of nine people, all affiliated either as family-volunteer, or as a medical-expert-volunteer at KHI.

The goal of this study was to reflect and report on how collaborative research practices affect people’s lives within a voluntary patient-driven research setting. An auto-ethnographic approach created an opportunity to report on different (and previously unreported) life domains and to contribute to a more balanced and complete presentation of the dynamics and impact of co-produced health research.

## Methods

### Research team and description of the collaborative process

The work at Ketotic Hypoglycemia International (KHI) is patient-led and supported by medical experts (represented in the Scientific Advisory Board of KHI) from the fields of endocrinology and inborn error of metabolism. Research at KHI is either patient-initiated or suggested by the scientific advisory board (SAB) and subsequently discussed with the parents at KHI. This is a fully co-produced research project; the research team consists of parents and medical/academic experts. Throughout this project, DD assured that everyone was consulted in case people were less active, couldn’t access mails or attend meetings (for example due to periods of hospitalisation of their child, invasive life events). No decisions are made without a team discussion or consultation with all members of the research team.

The participants in our study exhibit a diverse range of educational backgrounds and working conditions, from full-time caregivers with high school diplomas to senior medical professionals and academic researchers (see Table 1).

In the analytical section, we describe how we conducted the analysis collectively. We use the participation matrix [31] to clarify our roles at all stages of the study and the GRIPP2 short form [32] to detail the full collaborative process. This information is provided in Additional file 1.

### Auto-ethnographic study within a constructivist framework

Auto-ethnography is a qualitative research method where the researchers use a form of ethnography that applies self-reflection in writing-up and exploring a researcher’s anecdotal and personal experience [33]. The researcher reflects inward and on their own actions and observes themself in a particular role. Instead of relying on others to study the social reality at KHI (and therefore claiming someone else’s voice), we decided to study *society* through ourselves, delving further into the personal and professional impacts of being an active partner in collaborative health research. Using a methodology which intersects across multiple disciplines and overlaps with both writing, situational narratives, and research practices helped to create a collective understanding and common ground between the medical experts and the parents.

Social constructionism is a perspective which theorises that a great deal of human life exists as it does due to social and interpersonal influences [34]. It emphasizes the complexity and interrelatedness of the many facets of individuals within their communities. This theoretical lens aligns best with the values and philosophy of extreme citizen science, and mirrors the worldview underlying the work done at KHI and/or other co-produced health research projects.

### Data collection

Reflective conversations amongst the co-authors triggered by life events situated in the professional and personal lives of the members of KHI motivated us to undertake this work. We chose the mini-auto-ethnographic approach to allow us to focus on a specific subject of study [35]: a contextualized experience of the researcher during a particular time in the researchers’ lives. The specific topics used to generate the auto-ethnographic narratives were extracted from reflective conversations between the two first authors DD and AJ. The topics included (1) time investment, (2) power dynamics, (3) dealing with tasks outside the official job-related task load, (4) protecting personal boundaries, and (5) mental health. The suggested topics were shared with the co-authors, who all agreed on their relevance. These topics were translated into a number of probing questions, (at request of some co-authors) to guide their storytelling, and sent out via email. The volunteers each approached this task differently. Some wrote a coherent (lengthy) narrative, incorporating answers to all questions in one text, whilst others responded to each question separately. In one case, a medical volunteer answered the questions, and followed up with elaborations and reflections a few days later. With the consent of that person, these reflections were included to complement the initial data. Twelve parents and health care professionals, all members of the KHI organization, were invited to participate. Our study also includes a supplementary one-year follow-up status.

### Analytical process

We opted for interpretative phenomenological analysis (IPA) as an analytical approach [36]. IPA often deals with existential life events that impact how people think about themselves and their place in the world [37]. This research project was initiated following existential life events that were directly or indirectly related to being a volunteer worker at KHI. The aim of IPA is to explore how participants make sense of their personal and social world, and to extract the meanings particular experiences, events, states hold for participants. The approach is phenomenological in that it aims to explore personal experience and for that it considers the participant’s lifeworld and is concerned with an individual’s personal perception or account of an event, as opposed to an attempt to produce an objective statement of the object or event itself [38]. According to Larkin et al. the overall outcome for an IPA study should be “a renewed insight into the ‘phenomenon at hand’ - informed by the participant’s own relatedness to, and engagement with, that phenomenon” ([39], p. 117). Acting as data providers and data analysists, meaning making of the reported narratives was grounded in our lived experiences: we could question and discuss how meaning was attached to certain events as we had access to our own lifeworld.

The analytical process followed repeated cycles of two-level analysis: an analysis performed by the two first authors followed by a collective reflection and discussion of that analysis. This collective reflection was partially conducted in group session within KHI, via group email conversations and follow-up (informal) video and phone calls between the first author DD and some of the co-authors. The conversations amongst the research team enhanced the inter-subjectivity and co-constructed understanding of the data. The aim of these meetings was twofold; (1) to discuss the different rounds of analysis, and (2) to simultaneously work on and discuss the writing up of the paper. We chose this practice to stay true to how the KHI research team usually works together. The parents, who are also co-authors of this study, have worked on scientific papers and research via emails and video-meetings since 2020. They never met in person as a research group due to the global nature of the organization and a lack of funding to facilitate face to face meetings. The collective approach to the analysis reflected our understanding that discourse and meaning are intersubjectively and dialogically created. The analytical process did not have enough cycles to formally qualify as a collective auto-ethnographic (CAE) approach; however, the IPA approach allowed for rounds of interpretation and discussions typical for CAE [40] and turned a potentially semi-linear process into a collective, interpretative work.

In addition, a short supplementary 1-year follow-up status is provided after the presentation of the results of the above-described analysis.

### Ethics

Ethical approval is not required by Danish law for interview or text analysis. The project follows The Danish Code of Conduct for Research Integrity and is carried out in accordance with the Helsinki Declaration. Participants, in this case, authors of this paper, were informed about the purpose of the study and discussed potential risks and benefits with the first author(s). The data used in this study are provided by no other than the authors of the manuscript, who all approved of the manuscript.

The need for this study was triggered by events in co-authors’ lives, directly and indirectly related to their voluntary work at KHI. Although there was a shared need to conduct this work, this was the first time parents and SAB members engaged in auto-ethnographic work related to very personal themes. Also, this could evoke prior (negative) experiences. During the period in which the authors were composing their deeply personal confessions regarding their experiences at KHI, DD engaged in dialogues with all co-authors. These discussions focused on their experiences both within KHI and the process of documenting these narratives. One of these dialogues led to the decision to withdraw one of the parent narratives. This was a decision made by the parent; the draft narrative was withdrawn because it contained detailed and deeply personal accounts of a child’s medical journey – which was somewhat off topic and ultimately judged too sensitive to be published.

### Data availability

The datasets supporting the conclusions of this article are included within the article. The full narratives are stored on a secure server (University of Southern Denmark); anonymised versions can be consulted upon request (with the corresponding author).

## Results

Twelve volunteers were invited to share their personal narrative. Two invitees declined to join the project, citing a misalignment between their interests and expertise and the project’s scope. One parent volunteer wrote down their story realising it had shifted towards the (processing of the) traumatic journey of receiving a diagnosis for their child. In consultation, we decided not to include this narrative; the parent volunteer remained involved in the project. Nine volunteers participated: five medical experts and four parents of a child or children with IKH. Their characteristics are presented in Table 1. In the findings, we use the word *staff* to refer to family volunteers in KHI working on the day-to-day operations, as to reflect how family volunteers refer to themselves. No one in KHI gets paid for their work; hence, we have chosen to refer to them as “family-volunteers” to avoid confusion.

**Table 1:** Characteristics of those sharing their story

Medical-expert volunteer / Family-volunteer	Years affiliated to KHI	Gender	Country of residence	Education	Involvement experience	Working conditions
Family-volunteer	5 years	Female	Denmark	Master in Anthropology of Health	5 years	Senior Research Associate
Family-volunteer	3 years	Female	USA	Highschool	3 years	Full time caregiver
Family-volunteer	4 years	Female	UK	BSc	4 years	Health and Safety consultant
Family-volunteer	4 years	Female	USA	Highschool	4 years	Full time caregiver
Medical-expert volunteer	4 years	Male	Denmark	PhD	5 years	Clinical professor
Medical-expert volunteer	3,5 years	Male	UK	Clinician	3,5 years	MD
Medical-expert volunteer	2 year	Male	USA	Clinical Director	2 years	MD
Medical-expert volunteer	4 years	Male	USA	Clinician	4 years	MD
Medical-expert volunteer	4 years	Male	Denmark	Senior Executive	5 years	PhD

### Having found your people

The analysis revealed one dominant narrative that runs through all the stories as a red thread: having found your people. Everyone has their own unique KHI-(his)story; yet, being part of the KHI community, a community of like-minded people, is what all these stories have in common. All stories are situated in a shared *reality*: KHI is very much part of their lives, for better and for worse. All stories contain testimonies expressing a feeling of *home coming*, having found a group of like-minded people, a *fellowship*.

Parents write about having found a group amongst who they feel safe, understood and supported; a community where they feel `home´ amongst people who, like them, relentlessly pursue a better life for their child(ren). The latter is these parents’ greatest motivation, it is what keeps them going, even though it is at the cost of time spent with their children / family. The sacrifices made to contribute to KHI are difficult to share outside KHI, it puts them at risk of judgement and dismissal. The medical experts have found a niche in the hypoglycemia-world where they can make a difference, support parents; they have found *their people* in the KHI Scientific Advisory Board.

The staff and team at KHI understand the struggles of KH and while each member of the direct staff has their own medically complex child and ways that KH affects their daily lives, they understand and stand with me and my son and my family in this journey. KHI has helped in ways I never thought possible and have allowed me to find strength when seeking diagnoses for myself which are allowing me to embrace myself and my skills and attributes more and more each day. [P4]

Despite physical distance, different time zones, different nationalities, and native languages, there is a sense of cohesion, unity, and belonging build on a shared purpose. Albeit sometimes implied, the stories are all situated in a shared (virtual) *reality*, a place where finding answers to medical questions related to IKH is one’s life’s pursuit. This is what enables the medical experts and parents in KHI to free time and generate the strength to work at KHI.

The personal experiences (e.g. physical and or psychological trauma due to repeated under- or untreated KH attacks, diagnostic odyssey, …) and medical lingo shared between the members of KHI are experienced *foreign* to others, and potentially misconceived by those outside KHI. Being part of this community and pursuing this shared ambition is unquestioned within the community; it is a reality that does not extend beyond KHI.

These descriptions referring to the tight bond between fellow-members, the feeling of belonging, and the shared understanding of the dedication to the overarching goal, are significant of a tribe: a tightknit social community linked by *religious and or blood* ties with a common culture and language. The strong feeling of belonging and finding *your people* might also cause people to feel like they cannot leave or cannot say no to tasks; KHI is very much part of their lives, for better and for worse.

The four other identified narratives are presented separately, although they partially overlap with and all are grounded in the dominant narrative of *having found your people*.

### Invested time

No one is being paid for their services; all are volunteering their time to KHI. The stories illustrate that all feel pressured to contribute their time or to be flexible as to how and when they dedicate time to the organization. People give different reasons for the perceived pressure: the urgent nature of the requests (e.g. medical emergencies requiring support, last minute opportunities; Box 1 quote 1), the personal investment and, resulting from that, the self-inflicted moral pressure (Box 1 quote 2), the global nature of the organization and subsequent inconveniences (e.g. messages entering your inbox at all hours of the day/night; Box 1 quote 3), the collaborative nature of the work (e.g. many different people contributing to the same piece of work, creating different versions to review / approve), or wanting (or feeling social and individual pressure) to compensate and accommodate fellow-members’ limited availability (Box 1 quote 4).

**Box 1:** Citations related to the theme **Invested time**

1. “Some of the issues that I have spent time on in relation to KHI have furthermore been of an urgent nature, so postponement have not always been an option” [ME2].
2. “I consider the time I invest weekly as never enough. I wish I could give more on average. Our children deserve more. Our organization deserves more” [P2].
3. “Working globally, means working at all hours of the day” [P1].
4. “Working with volunteers, means being grateful and flexible around their time, while I always try to fit in after their needs and availability” [P1].
5. “It’s a balancing act of my job, my children’s KH, and non-KH medical needs” [P3].
6. “it really is a delicate balance, but it does allow for lining up with my son?s appointments. It?s a weekly jigsaw puzzle and it is often a tricky one” [P4].
7. “I’m stressed and exhausted, but my children need me to keep pushing for research and support so that someday they can live more normalized lives” [P3].
8. “This is something that I feel I have to do in my own time. Uncompensated time. Not part of my job expectation” [ME3].
9. “...have discretion over how I spend my time. Accordingly, I have chosen to devote time and effort to activities that are personally meaningful. If I were still working full-time and had my former workload and responsibilities, it would not be possible to do these things” [ME5]
10. “The additional work conducted over and above clinical work is unpaid and hence it?s difficult to reduce clinical work which may not be financially viable for everyone” [ME4]
11. “as funding has become scarce and extremely competitive, clinical work has become busier, there is the problem of demands of patients who are misinformed/confused, misinformation on the internet, health inequities, insurance challenges (US), etc.” [ME5].

Parents mention that time dedicated to KHI competes with time they need to attend to the medical and non-medical needs of their children, their daily jobs, and their partner (Box 1.5-7). Medical experts often consider working with patient organizations as part of their job and their professional responsibility as a medical expert. However, their job description does not include working for patient organisations, and the lack of formal recognition of this work by their employer has implications for their time and resources spent at KHI (Box 1.8) and their salaried position. One medical expert explains that they are at a point in their career, where they can financially afford to work in a reduced capacity which allows them discretion over how to spend the remaining (unpaid) time (Box 1.9). This is a personal decision which bears consequences; something noted by another medical expert (Box. 1.10). In addition, the medical experts explain that the medical profession and the practice of medicine has become more complex, both in regard to research funding, competition, time, health inequities, insurance challenges (US) and misinformed patients (Box 1.11), and therefore continuously lessens time and headspace to be spent outside the paid (hospital) position.

### Invested work

KHI is an international, non-governmental organization; the list of tasks associated with keeping such an organization afloat is long and diverse. The resources, in terms of experience, knowledge, skills, competences, network, that members contribute to KHI are different.

The medical experts contribute medical expertise and knowledge and take on tasks that are aligned with their daily professional activities for which they are medically trained: providing scientific expertise in inborn error of metabolism, endocrinology, and paediatrics (e.g. Box 2 quote 3). The medical experts categorized their contributions to KHI as non-renumerated *work*. Using the word *work*, they refer to work identical to tasks and activities performed as part of the paid professional medical role. In addition, some of the medical experts acknowledge that being part of KHI, obtaining experiential knowledge from the parents, exchanging knowledge with fellow medical experts, and contributing to novel research, benefits them and their clinical practice. It gives them the necessary input to improve the quality of care they and their colleagues offer on a daily base to patients with IKH (Box 2 quote 4). One could consider this as a return on invested voluntary work.

**Box 2:** Citations related to the theme **Invested work**

1. “When we united the experts in KHI, we realized that they didn?t have time or funding to start and lead research projects without additional manpower. We quickly realized that the families’ skills in KHI combined could make up for what would have been a PhD student’s job.” [P1]
2. “The publications on ketotic hypoglycemia only came into reality thanks to the networking that KHI led, uniting families, metabolic experts and pediatric endocrinologists across the globe. KHI has also done a tremendously great effort on the social media to inform about the disease and has hosted a great webinar conference” [ME1].
3. “attending meetings as a medical expert, meeting and educating the families on specific diseases and in your case ketotic hypoglycemia.” [ME3]
4. “I learn so much at each meeting from the families that I feel it is essential to my own personal development as a physician and that it allows me to improve the quality of care I offer” [ME3].
5. “With no leadership skills, or any knowledge about running an NGO, I started KHI with the support from scientific mentors and a big brother who had all of the skills I didn’t have in regards to communication, visual identity, graphical skills and networking skills” [P1].
6. “to enhance my skills and learn and grow as we carry out the work of KHI” [P4]
7. “being able to be a part of keeping everything moving forward and organizing the team and seeing the achievements and the outcomes, motivates me to continue to invest my time” [P4]
8. “Within months I was asked to step away as staff because I wasn?t doing enough. I was heartbroken but of course, I agreed because the work KHI was doing was too important for me to be slowing it down. Months later when an organizational restructuring occurred I felt blessed to be invited back, and I am proud of the work we are doing today” [P3].
9. “The medical experts can leave, and we will not be able to replace them”; whilst considering themselves “as [families are] replaceable” [P1].

Parents of (a) child(ren) with IKH affiliated with KHI come from all walks of life; some have a background in healthcare (e.g. nursing, psychology), others do not, some are familiar with research by education or profession, others are not. The parents contribute on a KHI needs’ basis in a more versatile and non-selective way compared to the medical experts. They take on *any* task that needs doing, for example administration, keeping KHI’s agenda, social media, literature searches, editing of papers for publication and funding applications, social media, organising conferences, KHI finances, etc. As such, the stories of the parents and medical experts illustrate that each group of volunteers (parents and medical experts) performs different tasks (Box 2 quotes 1, 2): parent volunteers take on any task whether they (think they) have the skills to do so or not – if necessary, they will learn on the job, medical expert volunteers perform tasks in line with their professional background and skills.

Participant 1 talks about their steep learning curve in the early days of the organization, when they learned essential skills required to do research and establish an NGO, by surrounding themselves with scientific mentors and turning to a family member highly skilled in communication, visual identity, and networking (Box 2 quote 5). Another parent shared a similar story of a steep learning curve, yet, with a different tone: working at KHI had given them an opportunity to learn and grow, and seeing the outcomes of their work motivated them to continue to invest their time (Box 2 quotes 6, 7). This parent volunteer saw this personal development as gains from contributing to KHI.

The differences in skills and competencies needed and relevant to research activities amongst parents had caused problems in the past with some parents only wanting to include parents with a medical or academic background. The organization went through a phase where there was debate about how they should present themselves, and how the volunteers of KHI should organize the activities, including guidelines about who could take part in research related activities (Box 2 quote 8). These fundamental questions about who *can* contribute, and who is *an asset* to the organisation, becoming a research organisation, split opinions. Eventually irreconcilable differences led to parents leaving the organisation. This had scarred some parents, who had left and returned; yet the newly found values and beliefs that *all parents* – regardless skills or professional background – could partake in (research) activities at KHI clearly resonated in the parent stories.

The differences in type of resources offered by the different members of KHI affects how these people perceive their contribution and those of other members of KHI. A parent talks about the invaluable contribution of experts, stating that the experts are irreplaceable, whilst considering themselves - families - as replaceable (Box 2 quote 9).

### Working together – power dynamics

The narratives also shed a light on the relationships between the different members of this tight community. The shared objectives are what ties the members of the group together, yet, personal and professional lives affect *how* the group works together, how they interact, and how they perceive these interactions and collaborative activities. The stories told are testimony of huge respect, warmth, and kindness members have for each other. This allows for medical experts and parents to work together side-by-side, feeling valued (Box 3 quotes 1–4).

**Box 3:** Citations related to the theme **Power dynamics**

1. “I feel there is a good balance, and the staff team works well together with respect and understanding. The staff work hard to work to resolutions when different topics are discussed in meetings, everyone is respected even if opinions differ and always try to understand where each other are coming from” [P4]
2. “When working with family organizations I believe we are all equals working together to improve the lives of the children we are responsible for. I enjoy the open conversation and feel very comfortable working with families in citizen science projects” [ME3]
3. “Working with KHI has been very exciting and motivational that provides platform between clinicians/ researchers/ families to share ideas and working together” [ME4]
4. “The power dynamics from inside KHI seem imperceptible to me, I see the scientific advisory board working with us and listening to our ideas when many in the medical field wouldn’t or haven’t” [P3]
5. “The normal patient/doctor/HCP relationship have in KHI been much more fluid than these relations ships traditionally are” [ME2]
6. “Initially when we were starting up KHI it was a more traditional relationship of me mentoring a ‘patient’ that wanted to create a patient organization. It very fast became a much more equal relationship where I actually started learning from P1. I personally have learned a lot [about citizen science] I can take back to my daytime job”.
7. “I consider the time I invest weekly as never enough. I wish I could give more on average. Our children deserve more. Our organization deserves more” [P2]
8. “This is the only job in the world that I can imagine outside of being a parent where you feel like you have your children’s future in your hands” [P3].
9. “The potential delay in new papers, new studies, and new collaboration for our kids” [P3].
10. “We are pleading for their time, their knowledge, and their volunteer dedication” [P1].
11. “The medical experts can leave, and we will not be able to replace them” [P1].
12. “It is essential to my own personal development as a physician and that it allows me to improve the quality of care I offer. For me this is a win-win situation” [ME3].
13. “In the initial phase parents were unaware of the time you need to spend, not only for the family representatives, but also for the physicians involved” [ME1].
14. “They are saving children’s lives, with their dedication to KHI” [P1].
15. “Medicine is an evidence-based science - right until they can’t explain it. KHI has blurred the power dynamics of medicine and research” [P2].
16. “Medical knowledge is overwhelming. It is unrealistic, therefore, to believe that physicians everywhere will have the knowledge, expertise and time to be able to correctly diagnose and properly manage every disorder that they encounter” [ME5].
17. “The parents offer a true perspective of what it is like to deal with medical complex children and how medicine and research needs to accept a change in the God complex that has overshadowed medicine for so many years”. [P2]
18. “It makes it difficult to trust ourselves and others again” [P3].
19. “It makes it hard for parents to step forward to work in an organization like KHI” [P2].
20. “When I know a different side of a person, such as their dedication to research and improving the lives of other families whose children have the same condition, it makes our medical interactions much more meaningful. It changes the trust dynamic and allows us to be more forthright with each other without offending each other” [ME3].
21. “Identify those questions and projects based on the most urgent needs of the families” [P1].
22. “I believe for example that the authorship of papers in the citizen science arena should be based on who came up with the idea, who drove the project, who did the work, NOT who is the doctor.” [ME3]
23. “How do you encourage and empower patients and parents and carers to work with medical experts. KHI has a delicate balance to play in ensuring the integrity of our research, but also as leading supporters of patient centred research and care!” [P2]
24. “Feeling appreciated is probably one of the best ways to improve our mental health, and parents are very appreciative of what we do” [ME3]
25. “KHI has only improved my well-being and my ability to keep fighting for answers. Because it is a safe haven, a place where you can find like-minded people, a place to fight for answers” [P2].
26. “Even if you feel you can cope because of the gratifying nature of the jobs the real question to ask: can your family cope and here it becomes more complicated. Ensure that you have not only a dialogue with yourself about your mental health but also involve your family and their wellbeing” [ME2].
27. “With a hope to find answers for my son, I started KHI. Little did I know that my desperate search for answers and a cure for my children, became what I now realize as one of the biggest burdens I have ever laid upon my family and my children” [P1].
28. “My daughter’s medical needs are such that in the 9 years of her life we have never slept more than two consecutive hours this is a burden on anyone’s mental health and well-being” [P3]
29. “The trauma we have experienced as a family has led to two of my three children being diagnosed with PTSD, and if I’m honest I would hazard a guess they aren’t alone; though we haven’t had time to seek treatment ourselves” [P3]
30. “I have had to limit my participation in some of the activities to protect my privacy” [ME1]
31. “I am getting better at determining when I need to say no to something, how to ask for enough time to get the task completed without feeling pressure” [ME3]
32. “There is a need for funding to support the members of the organisation that contribute to develop research activities. The funding will also enable clinicians to manage their time efficiently and prevent burn-out” [ME4].

In trying to describe the power dynamics and relationships between parents and medical experts, they compare what they have (created) at KHI with the other relationship they often still have outside KHI, namely the patient-doctor relationship in a clinical setting (Box 3 quote 5). The stories gave insight in how their relationships had evolved and revealed elements that influenced the power dynamics between medical experts and parents. At KHI, the parent’s position changed from the one being taught by the medical expert in a consultation setting, to an equal partner; and on the other side, the expert started to learn from the patient (Box 3 quote 6) whereas they used to be the sole expert in the room.

#### Being personally invested

Parents shared that they feel they cannot leave the organization and feel guilty of not dedicating enough time and resources to the organization (Box 3 quote 7). This organization, and contributing to its cause, offers parents a lifeline and tangible hope to improve their child’s life (Box 3 quote 8). Parents put huge pressure on themselves, fearing that not dealing with the KHI tasks at hand (fast enough) could result in delay in papers, studies, and collaborations and eventually obstruct a breakthrough in the discovery of a treatment for IKH (Box 3 quote 9). This constant (self-imposed) pressure and internal conflict was not reported by medical experts.

#### Resources contributed and gains obtained

Knowledge regarding a highly specialised, narrow field of research in a rare disease gives you authority and makes you a rare resource. Parents are immensely grateful for the medical experts for joining KHI and offering time and resources; parents feel indebted to them (Box 3 quote 10). Parents consider the medical experts as irreplaceable (Box 3 quote 11). Whilst they have a very rare and valuable resource to offer, medical experts contribute skills and competencies obtained for and used in their daily jobs. Albeit unique in the bigger picture, their resources are readily available and common use to the medical experts. In addition, in meetings with parents they learn things unique to their interactions at KHI which the medical experts consider essential to their personal development as a physician (Box 3 quote 12). Parents have had to learn many of the skills needed to complete tasks at KHI, including tasks not related to research e.g., how to run an NGO and making budgets. While the contribution of the medical experts might be considered an extension of their daily jobs, it requires more than the time witnessed by parents; something parents perhaps cannot grasp (3 quote 13). These imbalances, for example bringing skills obtained as part of a paid job versus acquiring new skills (in your own time) to be able to contribute to KHI, were new to many when analysing the stories. This led to both parties supplementing their stories; for example, medical experts explained how delivering towards their KHI tasks extended beyond work visible to the parents (such as consulting colleagues, exploring new medical avenues).

#### The (lack of) power of the medical system and medical knowledge

Parents and medical experts bring different knowledge to KHI: evidence-based medicine (and related skills and expertise) versus personal experiences of living with IKH. At KHI, parents have actively approached medical experts to join the organisation in search for medical expertise. Parents mention in their stories that the contribution of the medical experts is much more important compared to theirs. First, their knowledge contribution to the organisation is saving children’s lives (Box 3 quote 14) and secondly, because of the scarcity of these goods. On the other hand, the reason that KHI was founded, was because there were no answers to the (medical) questions parents had (Box 3 quote 15), somehow exposing the finitude of (their) medical knowledge. Parents, as well as medical experts, share their reflections on this duality of lived experience and medical knowledge in their stories. One medical expert explores the concept of medical knowledge, acknowledging that medical knowledge is overwhelming, creating unrealistic expectations for physicians to have the needed knowledge, expertise, and time to be able to diagnose and manage every disorder they encounter (Box 3 quote 16). A parent shares a similar reflection, noting the impact of parents’ insights in dealing with medically complex children, and how medicine and research must accept a change in the “God complex” that has overshadowed medicine for years (Box 3 quote 17).

#### The (prior) patient – doctor / healthcare system relationship

Parents all have a history with the healthcare system; not infrequently, this includes bad experiences with health care professionals who do not believe them or accuse them of hurting their children. This journey towards a diagnosis does not leave the parents unscarred. These prior experiences make it difficult for parents to trust themselves and others again (Box 3 quote 18). The fight they have had to endure on behalf of their child, after having been dismissed by doctors, traumatized by accusations of a number of unspeakable child safeguarding concerns, makes them vulnerable (Box 3 quote 19).

Parents are acutely aware that, due to their work within KHI, they have “direct access” to medical experts other parents might not have. They constantly worry that they might be inappropriately using or accessing medical knowledge and expertise of the Scientific Advisory Board of KHI. Likewise, medical experts also acknowledge that working with certain parents in collaborative research projects changes the patient-doctor relationship during clinical practice (Box 3 quote 20) and not seldom they questioned how to deal with that.

#### Equal partners in research production

When parents and medical experts talk about their collaboration in research, parents describe that they are in charge of certain elements of the research process, and feel they have the power to identify research questions based on the urgent needs of the families (Box 3 quote 21). Medical experts state that they see no reason for not acknowledging parents’ roles in the research process, via, for example, authorship (Box 3 quote 22). It is in their collaborative research efforts that they see a transformative role for KHI (Box 3 quote 23).

### Personal boundaries and mental health

The time and work invested and the power-dynamics at play in the relationship between parents and medical experts affects these people. Being affiliated with and working for KHI brings pleasure and joy, and creates meaningful relationships (Box 3 quotes 24, 25). The work they do for KHI has implications beyond their own lives. They are all adults, making their own decisions, fully aware that their decisions affect their own life and the lives of their loved ones (Box 3 quotes 26, 27). There is the time committed to KHI at the cost of time spent with family, the irregular working hours at KHI, the burden and mental stress related to (medical) emergencies shared, and pressing tasks. These are all things described by both family volunteers and medical expert volunteers. For the parents, there are also other elements impacting their mental health and wellbeing: the care for their child (Box 3 quote 28), the uncertainty about their child’s future, past poor experiences with the health care system (Box 3 quote 29), and financial implications of the complex medical needs of their child.

Many of the parents and medical experts have shared their struggles with setting boundaries and safeguarding their mental health with varying degrees of success. Their stories include issues with not being able to say no to tasks for KHI, lack of personal boundaries on the workload, panic attacks and night terrors related to work situations, and burn-out. Some managed to install protective measures (Box 3 quotes 30, 31) and/or sought professional help. All stories indicate that this remains something to monitor, and be mindful of, which in the end requires a more structural solution in terms of securing funding to prevent burn-out and other negative effects (Box 3 quote 32).

### One-year follow-up status

It has been one year since we collected and analysed our stories. This is unfunded work, conducted on top (or alongside) of other work and personal commitments of parents and medical experts which often leads to lengthy research processes. Since then, many things happened in the professional and personal lives of the authors. We feel morally obliged to report on some of these (this is in no way an exhaustive list). The chairman of the scientific advisory board (SAB) had to withdraw from their commitments in KHI due to the immense work-pressure both at the hospital and KHI. A SAB member had to withdraw from the board due to the work pressure but found their way back after re-arranging their paid work to be able to dedicate their time fully to the patients suffering from IKH. A personal (unedited) reflection is shared in Box 4.

**Box 4:** Personal reflection of first author DD, towards the end of the writing process of this paper

This project, bringing the emotional and physical impact of voluntary collaborative research activities to the front, made me reflect upon the mind-blowing amount of hours we have dedicated, and the great people who had to cut ties underway due to the unspoken pressure we undeniable inflicted upon ourselves and each other working with a mission so close to heart.
Most caregivers are not fine; this is not new and not unique to our group of parents. There is a plethora of research, long ignored by policy makers and health care professionals [41, 42]. Our seemingly endless energy and willingness to take (the lead) on tasks might be a sign of just how desperate we are to create hope for a better future. This comes at a price, as each dedicated hour to research tasks, is another hour away from our families or another hour not spent on much-needed rest. Looking back, I see a relation between hours spent at KHI and my children’s condition; the worse they were, the harder I chose to work, as I felt I was responsible for finding the answers needed to fix them. The more they needed me, the less I was present. When working with patients or families in research, we must be aware of the physical and emotional burden it is to be a caregiver (or patient), and how these circumstances can create an unhealthy life-balance.
Despite all the hours, and the considerations, I would still encourage you to dive into patient-driven research. Just remember to take care of yourself, your family, and your research-team underway to keep the fiery souls from burning out.

Our work is an expression of passionate physicians wanting to make a difference and desperate parents working tirelessly to create hope. Our KHI organization continues the work in a strengthened position, constantly trying to incorporate the learning from the present study in daily practice and future planning.

## Interpretation and discussion

Patient and public involvement and engagement in research (PPIE) and especially co-produced health research has gained momentum in the last few years as an emerging research field [43]. This paper contributes to a gap in the literature and opens the debate on the much underreported adverse effects of patient-driven and or collaborative health research. Exploring the power-dynamics, roles and responsibilities of parent volunteers, and medical expert volunteers in a non-governmental patient organisation, we identified two main adverse effects: (1) the mental and physical effects on the volunteer and their loved ones of time, work, and other non-compensated resources dedicated to a voluntary organisation; and (2) the two-sided coin of being part of a *tribe*: the comfort and solace, and (social) pressure.

### The toll of voluntary work

Lack of time and appropriate skills are often-mentioned barriers for both patients and researchers to work collaboratively in health research [16]. In the case of KHI, both parents and medical experts go above and beyond to learn new skills and dedicate time to the work and research activities at KHI. The lack of time is known to be a key obstacle for researchers; PPIE activities are still an extra task to be integrated in the pressed time-schedule of researchers and *informal* work related to PPIE (for example, building and maintaining relationships with patients, preparatory work) often goes unacknowledged [21, 44]. However, the physical and or psychological impact, due to boundaries being crossed, have not been reported earlier. The burden of responsibility and duty, as well as time and financial burdens of patients and relatives have also previously been reported [45]. In a survey amongst 147 Swedish informal caregivers, time was indicated as the most common perceived obstacle to become actively involved in health research [46]. Others reported that patients experienced stress, fatigue, and guilt of not contributing enough while incorporating their patient engagement activities in their daily lives as they competed with existing priorities [47]. An American Patient Engagement Group (PEG) also reported that participation in studies could become overwhelming, depending on what was happening in their personal lives [48]. Both papers reported that this burden was somehow lessened as the researchers with whom they collaborated showed consideration for what was going on in participants’ lives beyond the research setting. A supportive and understanding setting helped patient partners to restore balance.

### The sense of belonging and being part of a tribe

The community at KHI was indeed perceived as supportive and understanding. More so, the closed-knitted community created a safe place where, through common language, experiences could be shared that could not be understood by non-members of the community.

We found that the tight-knit community at KHI could be described as a tribe: a small community of like-minded people committed to a shared goal, with a shared language and a strong sense of solidarity. Putnam (1995b) defines social capital as “features of social life—networks, norms and trust—that enable participants to act together more effectively to pursue shared objectives” (p. 665). The (online) community of KHI could be considered social capital. This sense of community and belonging created positive impact on its members’ personal and professionals’ lives: feeling safe and understood, exchanging and obtaining knowledge, and access to a support network. The other side of the coin is that the sense of belonging and responsibility towards community members increased the risk of disrespecting and crossing one’s personal and professional boundaries, if not causing, at least not protecting the members from the reported physical and mental harm. The tribal community might create a false sense of safety when people cross their own boundaries because of their community membership. Altruistic motives and personal salience are often mentioned as the driven factor for patients to engage in collaborative research activities [48, 50]. However, as these findings show, they might also lead to overcommitment and unhealthy situations.

### Knowledge and skills in collaborative research

These narratives revealed that patients perceive themselves as less knowledgeable and less important partners. PPIE and collaborative health research practices is about sharing and producing knowledge; recent studies have drawn attention to the epistemic complexities involved in collaborative practices. The concept of epistemic injustice refers to an injustice done to individuals in their capacity as knowledge bearers, reasoners and questioners, in which their ability to take part in epistemic practices, such as giving knowledge to others (testifying) or making sense of their experiences (interpreting), is weakened [51]. In our study, we see parents devaluing their knowledge – and as a result themselves. They consider their experiential knowledge or self-taught skills as less valuable and less credible compared to the medical knowledge the medical experts contribute. The parents have *learned* over the years that they cannot trust their own (experiential) knowledge. In the diagnostic journey, some parents had been accused of harming their child, whilst others had been received with disbelieve reporting symptoms. This could be considered an example of epistemic exclusion; an infringement on the epistemic agency of a knower that reduces her or his ability to participate in a given epistemic community [52]. In addition to these self-inflicted epistemic credibility issues, there is the epistemic privilege all members of the medical science field possess [53]; the scarcity of medical experts dedicated to IKH adds to the special status that was attributed to them. The combination makes that parents tend to compensate their inferior epistemic position with taking on a disproportionate amount of non-epistemically-loaded tasks, such as running the social media profiles, administrative and financial chores, and feel indebted to and eternally grateful for the medical experts’ contribution.

Also, within the group of parents at KHI, there was debate about who could contribute to epistemic practices: some parents considered it paramount to have research knowledge and evidence-based research skills, “being knowledgeable enough”, to partake in research activities. Training for *lay people* prior to engaging in research activities has been reported as both a facilitator and a hindrance to meaningful PPIE [21, 54, 55]. This debate will continue to trouble collaborative practices when instrumental goals are the primary driver for PPIE. At KHI, a decision was made not to exclude anyone from any activity.

### Power imbalances in research relationships

Notwithstanding the parents’ perceived inferiority, parents at KHI are in control of the running of the organisation and initiation of (research) activities; they all stressed that they acted as equal partners in the organisation and research activities. More so, the diversity of educational and professional background the parent volunteers, despite varying levels of formal education, were able to contribute significantly by leading research efforts from their community, thereby alleviating some of the burden on the scientific volunteers. This collaborative approach highlighted that research skills and lived experiences together enrich the research process, making it more inclusive and ensuring that all participants are valued for their contributions. Therefore, it is worth further investigating whether actual roles or perceived value feeds into power imbalances in patient – researcher partnerships.

Another relationship that might introduce power-imbalance, is the (potential) ongoing clinical relationship, and how to shift between the patient/caregiver – doctor relationship to a research relationship as partners. The researchers and clinician volunteers in this study wield substantial influence as gatekeepers to the proper care, safety, and support for children globally, including those cared for by the caregiver volunteers in KHI. Given the scarcity of healthcare professionals who understand IKH, these experts are highly valued and often esteemed. Establishing a collaborative relationship with them requires caregiver volunteers to navigate a delicate balance: advancing research and revising medical literature while preserving the essential doctor-caregiver/parent relationship crucial for the immediate safety of the children involved. This relationship adds to the complex dynamic between parents and medical experts and emphasizes the critical need for thoughtful negotiation in collaborative endeavours.

### Ethics of care

Instead of questioning “the feasibility of equal involvement of all citizens” due to “the unintended negative consequences” particularly experienced by “vulnerable and disenfranchised groups” [56], we advocate for an ethics of care in collaborative and patient-driven research [57]. We need safeguarding guidelines for collaborative health research, so we can deal with dilemmas in responsibility to care for co-researchers (patients, members of the public, and academically trained researchers), and instil self-care and existential safety for an ethical collaborative research practice. The framework of ethics presented by Groot and Abma [58] offers a heuristic guide to reflect on ethics in researchers’ daily practice. Working in the bio-medical field of IKH, reflective work is new to many research partners. We suggest introducing collective auto-ethnographic practices as an aid to perform the ethical work. There is a close association between qualitative heuristics and classical cultural anthropology and ethnography [59] and although new to many medical experts, as human beings we are constantly immersed in heuristic processes [60].

### Strengths and limitations

This collaborative research stems from a voluntary organization, founded by and for patients, where medical experts are invited in. Therefore, both the studied setting as the team conducting this research are different from other PPIE research, where patients are ‘invited in’ [61]. Further, our study was limited to KHI and its volunteers, therefore it is not necessarily a representative sample of the citizen-science community. Therefore, our reported impact of collaborative research will differ from other work known as PPIE.

As an organization run by volunteers, both patients and medical experts, we wanted to use and work with our own experiences of collaborating in health research to contribute to the limited knowledge available about unwanted or undesirable consequences related to co-produced health research. Using auto-ethnographic data, we “re-introduced the self as a methodological resource” [62]. We use this methodological tool to assist in the process of knowledge democratization, legitimizing multiple types of knowledge, while understanding knowledge as a situated product, relational practices of representation, rather than a neutral, context-independent foundation [63]. With our study, we take the auto-ethnographic approach into the field of medicine.

Due to fear of a lack of anonymity in the published paper there could be limitations to the true veracity or completeness of the self-reported experiences, as the willingness to disclose might have been compromised. Another limitation was the non-participation of 25% of the invited KHI volunteers and the four KHI core members who resigned from KHI prior to this study.

## Conclusion

This auto-ethnographic study of co-produced health research in the patient organization KHI focused on time invested, work invested and power dynamics for participating parents and health care professionals. PPIE not only led to changes in power dynamics between parents and health care professionals, but also adverse experiences for both groups, including excess workload and struggles with maintaining boundaries and safeguarding their mental health within the prevalent resources. Our findings call for the need to secure and prioritize structural funding to accommodate patient-driven and patient-led knowledge activities, for example research infrastructure (i.e. multidisciplinary research laboratories, scientific instrumentation and technologies, data resources, and communication networks) and budget to buy in skills lacking in the team. Existing ethics frameworks could be used to advocate for ethical boundary work.

### Electronic supplementary material

Below is the link to the electronic supplementary material.

Supplementary Material 1

## Data Availability

The datasets supporting the conclusions of this article are included within the article (Box 1–4). The full narratives are saved on a secure server (of the University of Southern Denmark); anonymised versions can be consulted upon request (with the corresponding author).

## Abbreviations

PPIE: Patient and public involvement and engagement
KHI: Ketotic Hypoglycemia International
IKH: idiopathic ketotic hypoglycemia
UK: United Kingdom
US: United States
