# Supplementary material for: An auto-ethnographic study of co-produced health research in a patient organisation: unpacking the good, the bad, and the unspoken
Source: Res Involv Engagem. 2024 Jul 23;10:76. doi: 10.1186/s40900-024-00609-8 (PMC11265487; doi:10.1186/s40900-024-00609-8)
Supplement: Supplementary file 1 — Supplementary Material 1 [file 40900_2024_609_MOESM1_ESM.docx]

Additional file: Detailed description of the collaborative process using GRIPP2 and Involvement Matrix.

Table 1: GRIPP2 Short form

| **Section and topic** | **Item** | **Reported on page No** |
| --- | --- | --- |
| 1: Aim of PPI in the study | This is patient-led research initiated by parents of children with idiopathic ketotic hypoglycemia (IKH), all affiliated with Ketotic Hypoglycemia International (KHI). The patient organisation was founded in 2020 by Danielle Drachmann, mother of (now) three children (two of them diagnosed with IKH). A patient-led collaborative research network forms the foundation of the organisation. KHI consists of parents of (a) child(ren) with IKH and medical experts (represented in the Scientific Advisory Board) from the fields of endocrinology and inborn error of metabolism. The mission of KHI is to enhance the understanding of IKH for the benefit of children, parents, and families who have been affected by IKH.  Research at KHI is either patient-initiated or suggested by the SAB and subsequently discussed with the patients at KHI. All research projects are conducted by a team of parents and medical experts. This research project was no different. It is part of the DNA of this research collaborative to have parent-patients in the driver seat. | Pg 8-9  Pg 10 |
| 2: Methods (description of the methods used for PPI in the study) | This is a fully co-produced research project. The research team consists of parents and medical/academic experts. No decisions are made without a team discussion, and consultation of all members of the research team. DD takes on the tasks to assure that everyone is consulted in case people are less active, cannot access mails or attend meetings (for example due to hospitalisation of their child, invasive life events).  In the method section we describe how we conducted the analysis as a team effort. To clarify roles throughout the study we completed the participation matrix. | Pg 10, 12-13, 34 |
| 3: Study results: Outcomes (the results of PPI in the study, both positive and negative outcomes) | This is patient-led research supported with expertise from medical experts and or academic scholars. In this case, a new methodology was introduced: auto-ethnography. DD was familiar with the methodology; her collaboration with AJ empowered DD to initiate a study using a methodology unfamiliar to the medical experts at KHI. In this case, an external (to KHI) methodological expert was invited to assist in executing a research idea. | Pg 27-28 |
| 4: Discussion and conclusions Outcomes (the extent to which PPI influenced the study overall). | All parents and medical experts that have contributed to this study are listed as authors/co-authors. It is difficult to describe to what extent PPI influenced the study: this is a fully co-produced study. This paper had not existed if KHI had not existed: the idea for this study stemmed from a parent; the findings and discussion got wrapped up because an academic expert led the analytical process and writing up of the findings and discussion; the analysis would not have been as authentic without the contribution of all members; the paper made it to the finish line because each and every one (parent and academic/medical expert) wanted this project to succeed.  This is an unfunded project which took as long as it needed; we had not set ourselves a deadline. At times we did not make any progress for weeks or months because people (parents as well as medical experts / academics) were otherwise engaged, overwhelmed, were needed elsewhere, and couldn’t dedicate time towards the study. Our findings are a good reflection of how we experienced this research process. | Pg 27-28 |
| 5: Reflections/critical perspective (critical comments on the study) | Analysing our data and writing up our findings had a serious impact on many of the (co-)authors. For some it had a therapeutic effect: power dynamics were discussed openly, perceptions of work and time committed towards KHI shared. For some it was an eye-opener, a wake-up call, as key life domains were out of balance, and they were forced to acknowledge and deal with this as they were writing it up.  The method used – auto-ethnography combined with collective analysis (simulating a collective auto-ethnographic process) – was new to some and therefore challenged the *usual* dynamics in the group (who was expert and who less experienced in the research methodology and topic of research). Team members had a different *feel* for the study at the start of the project: some were not convinced of the methodology used, others felt challenged by the idea of sharing their narratives. Now that we have written up the findings and discussed how our findings could contribute to new developments in the field of extreme citizen science, patient-led research and patient and public involvement and engagement, everyone is proud of what we produced and convinced that this is necessary and long-overdue contribution to the field. | Box 4, Pg 24 |

Table 2: Description of roles of all authors to clarify our ’method of PPI’

| Stage | | Listener  (is given information) | Co-thinker  (is asked to give opinion) | Advisor  (gives (un)solicited advice) | Partner  (works as an equal partner) | Decision-maker  (takes initiatives and/or decisions) | |
| --- | --- | --- | --- | --- | --- | --- | --- |
| Preparation | Research question |  |  |  | AJ, KBC, AC, HTC, BF, YOL, TP, JSP, PS, PT, JW | DD |  |
|  | Protocol |  |  | KBC, AC, HTC, BF, YOL, TP, JSP, PS, PT, JW |  | AJ, DD |  |
|  | Study design |  |  |  | AC, HTC, BF, YOL, TP, JSP, PS, PT, JW | AJ, DD |  |
| EXECU-TION | Data collection |  |  |  | AJ, DD, AC, HTC, BF, YOL, TP, JSP, PS, PT, JW |  |  |
|  | Data analysis |  |  |  | KBC, AC, HTC, BF, YOL, TP, JSP, PS, PT, JW | AJ, DD |  |
| IMPLEMEN-Tation | Write-up |  |  | KBC, AC, HTC, BF, TP, JSP, PS, PT, JW | YOL | AJ, DD |  |
|  | Dissemination |  |  |  | Work in progress | Work in progress |  |

Note: Initials of authors are used. Astrid Janssens (AJ), academic researcher, did not contribute with a narrative; was not member of SAB at the time of this study. Danielle Drachmann (DD), parent, contributed with a narrative. Kristy Barnes-Cullen (KBC), parent, did not contribute with a narrative; joined KHI at the very start of the project. Austin Carrigg (AC), parent, contributed with a narrative. Henrik Thybo Christesen (HTC), Medical expert, contributed with a narrative. Becky Futers (BF), parent, contributed with a narrative. Yvette Ollada Lavery (YOL), parent, her narrative turned out to be more a reflection on the trauma from the diagnostic odyssey, therefore, it was decided not to include her narrative. Tiffany Palms (TP), parent, contributed with a narrative.

Jacob Sten Petersen (JSP), Medical expert, contributed with a narrative. Pratik Shah (PS), Medical expert, contributed with a narrative. Paul Thornton (PT), Medical expert, contributed with a narrative. Joseph Wolfsdorf (JW), Medical expert, contributed with a narrative.
